# Bilateral sequential Ramsay Hunt syndrome in an immunocompromised adult: a rare entity

**DOI:** 10.1308/rcsann.2022.0133

**Published:** 2023-11-30

**Authors:** S Sheik-Ali, Y Jiang, H Nasef, E Sproson, O Tuohy

**Affiliations:** Portsmouth Hospitals University NHS Trust, UK

**Keywords:** Ramsay Hunt syndrome, Immunocompromise

## Abstract

Bilateral Ramsay Hunt syndrome is a rare clinical entity. We present a case of bilateral Ramsay Hunt syndrome in an immunocompromised adult, with sequential symptom onset. It is important to consider this as a differential diagnosis in patients presenting with bilateral lower motor neuron facial weakness.

## Case history

A 53-year-old female presented with a six-day history of left-sided otalgia to our ear nose and throat (ENT) department in October 2021. This was associated with a vesicular rash involving the left pinna and external auditory canal, with subsequent blistering. She also described reduced left-sided hearing. Some 48h following her hospital presentation, she developed unilateral left-sided facial weakness (Grade IV on the House–Brackmann scale), with the inability to close her left eyelid.

This was on a background of recent chemotherapy for Stage IV high-grade serous ovarian cancer, with lung, spleen, nodal, umbilical and peritoneal metastatic deposits. She had completed cycle five of six of chemotherapy (carboplatin, paclitaxel and bevacizumab) one week prior to her initial symptom onset.

**Figure 1:**
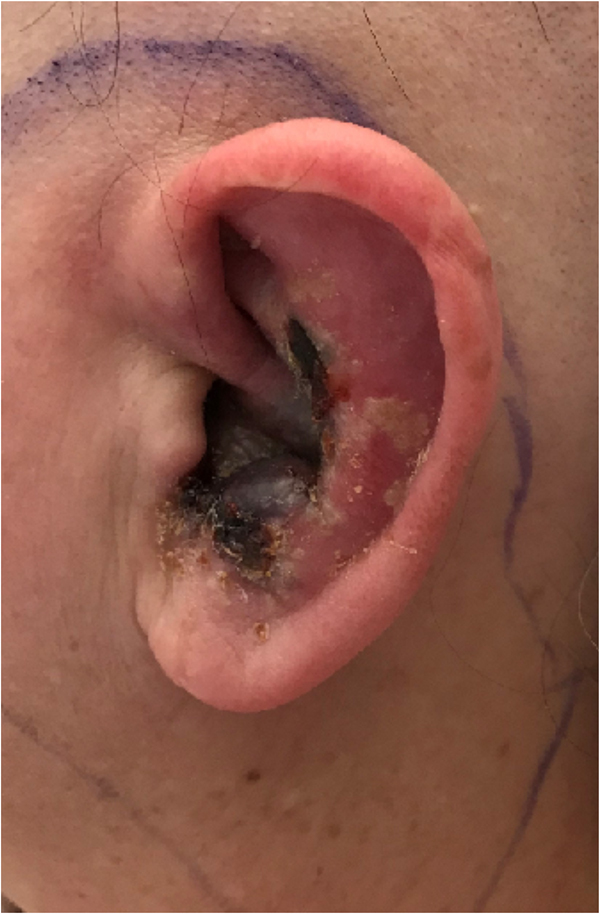
Left pinna

On examination, otoscopic and microscopic evaluation of her ears revealed an extensive vesicular eruption involving the left pinna and external auditory canal, with an associated necrotic focus in the conchal bowl (Figure 1). Weber’s test lateralised to the right ear and Rinne’s test was positive on the left ear, indicating left-sided sensorineural hearing loss. Cranial nerve examination demonstrated unilateral left-sided lower motor neuron facial nerve palsy – Grade IV on the House–Brackmann scale.

The patient was admitted for a series of investigations, with input from the ENT, neurology and oncology departments. Swabs from the left external auditory canal were polymerase chain reaction (PCR) positive for Varicella zoster virus (VZV). Serum *Borrelia burgdorferi* tests were negative for Lyme disease. Screening for HIV to exclude other immunosuppressant conditions was negative.

**Figure 2:**
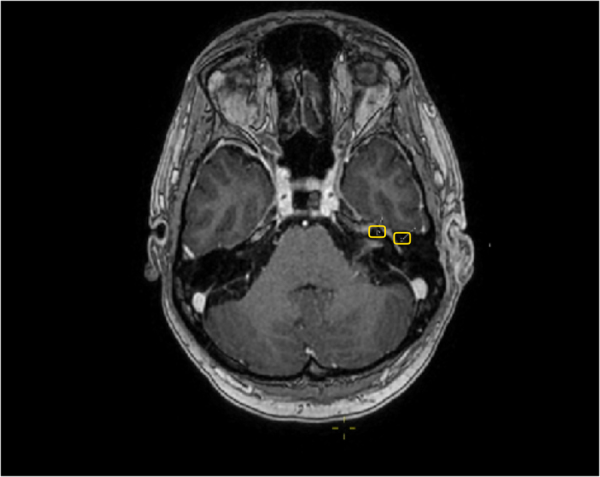
Magnetic resonance image of internal auditory meatus

A high-resolution computed tomography (CT) scan of her petrous bones with contrast was conducted in October 2021. This demonstrated subcutaneous thickening around the left external acoustic meatus without focal collection, with no other abnormality identified. Magnetic resonance imaging (MRI) of the head/internal auditory meatus (IAM) with gadolinium was performed in November 2021. This demonstrated asymmetrical hyper enhancement of the tympanic and mastoid segments of the left facial nerve and both IAMs (Figure 2). Her symptoms and investigations were thus consistent with a diagnosis of Ramsay Hunt syndrome.

The patient was treated with a 14-day course of 60mg (1mg/kg) oral prednisone. Intravenous aciclovir was given at a dose of 400mg five times a day for 14 days. In addition, she received a course of intravenous tazocin, oral ciprofloxacin and topical Gentisone drops. Given the inability to close the left eye, ophthalmology were consulted and she received eye care in the form of lubricants, false tears, taping and facial nerve rehabilitation exercises.

**Figure 3:**
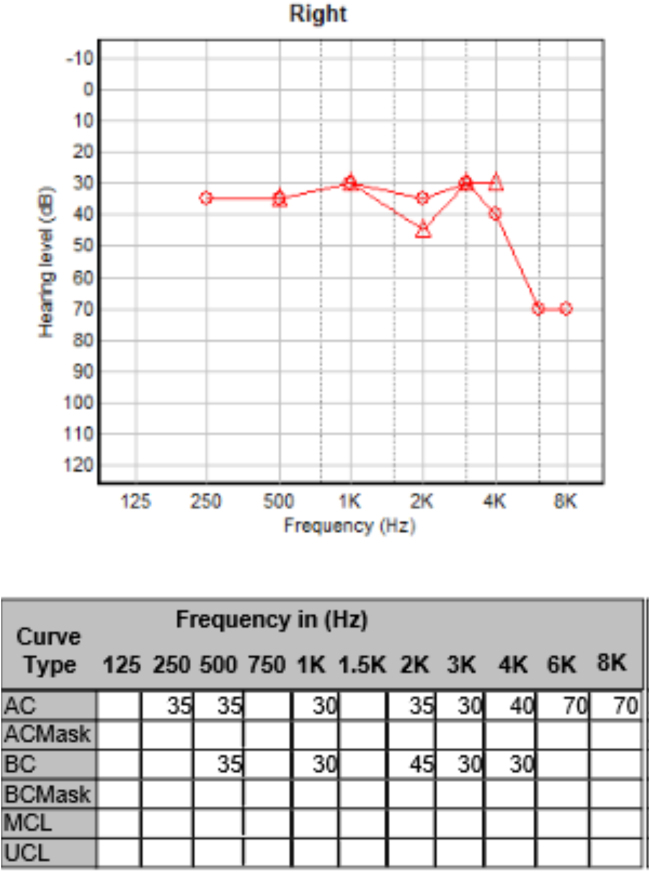
Pure tone audiogram of the right ear

Two weeks into her hospital admission, the patient developed a new right-sided lower motor neurone facial nerve palsy, graded IV on the House–Brackmann scale. This was symmetrical in nature, meaning she had developed a sequential, bilateral facial nerve palsy. Pure tone audiometry revealed mild–moderate sensorineural hearing loss of the right ear (Figure 3).

Given symptom progression, a repeat MRI head/IAM was conducted in November 2021. This demonstrated prominent bilateral cochlear and facial nerve bundles within the intra-meatal course. Given the patient’s background of metastatic ovarian cancer, a lumbar puncture was conducted to exclude a diffuse leptomeningeal process to suggest carcinomatosis.

Cerebrospinal fluid cytology did not demonstrate any malignant cells. Of note, a recent surveillance CT scan of the patient’s neck, chest and abdomen in September 2021 had not demonstrated progression of her underlying cancer, with falling CA-125 levels. Therefore, her symptom progression and investigations were in keeping with a diagnosis of bilateral, sequential Ramsay Hunt syndrome.

The treatment outlined above continued for a further week. Given the onset of Ramsay Hunt syndrome, the patient’s chemotherapy treatment was halted and she remained under disease surveillance. There was complete resolution of the vesicular rash involving the left pinna. There has been incomplete recovery of the bilateral lower motor neuron facial weakness, improving to House–Brackmann Grade III.

## Discussion

Ramsay Hunt syndrome is a facial nerve palsy that manifests as a reactivation of latent VZV in the geniculate ganglion, associated with an erythematous vesicular rash on the ear (zoster oticus) or in the mouth. The diagnosis is a clinical one – often presenting with otalgia, ipsilateral facial paralysis and vesicles in the external auditory canal. Other symptoms include tinnitus, hearing loss, nausea, vomiting, vertigo and nystagmus due to the vestibular–cochlear nerve being in close proximity to the geniculate ganglion.

The phenomenon of bilateral Ramsay Hunt syndrome with sequential symptom onset is rare. It has been reported in a case of poorly controlled diabetes mellitus.^1^ A case of asymmetrical facial nerve weakness as a result of Ramsay Hunt syndrome has been reported in an immunocompetent adult in China.^2^

In the early stages, Ramsay Hunt syndrome is sometimes indistinguishable from Bell’s palsy. Up to 14% of patients develop vesicles one week after the onset of facial weakness. Although rare, some patients develop a peripheral facial paralysis without ever growing vesicles in the ear or mouth, also known as zoster sine herpete. In these cases, VZV DNA can be detected on PCR in auricular skin, blood mononuclear cells, middle ear fluid or saliva.^3^

MRI is the imaging of choice, and increased abnormal signal on contrast-enhanced T1-weighted MRI has been shown to be positively correlated with facial nerve swelling.^4^

The use of a corticosteroid and aciclovir in combination has been found to induce a more complete recovery than the use of a corticosteroid alone, with methylprednisolone being the steroid of choice. A recent systematic review has also found that intratympanic corticosteroid may also potentially stimulate recovery in patients with Ramsay Hunt syndrome.^5^

## Conclusions

This paper describes a rare case of bilateral Ramsay Hunt syndrome in an immunocompromised adult, with sequential symptom onset. It is important to consider this as a differential diagnosis in patients presenting with lower motor neuron facial weakness.
